# Antibacterial and Antibiofilm Activity of Endophytic *Alternaria* sp. Isolated from *Eremophila longifolia*

**DOI:** 10.3390/antibiotics12091459

**Published:** 2023-09-19

**Authors:** Daniel J. Caruso, Enzo A. Palombo, Simon E. Moulton, Peter J. Duggan, Bita Zaferanloo

**Affiliations:** 1 Department of Chemistry and Biotechnology, School of Science, Computing and Engineering Technologies, Swinburne University of Technology, Hawthorn, VIC 3122, Australia; epalombo@swin.edu.au; 2 Department of Engineering Technologies, School of Science, Computing and Engineering Technologies, Swinburne University of Technology, Hawthorn, VIC 3122, Australia; smoulton@swin.edu.au; 3 CSIRO Manufacturing, Research Way, Clayton, VIC 3168, Australia; peter.duggan@csiro.au; 4 College of Science and Engineering, Flinders University, Adelaide, SA 5042, Australia

**Keywords:** endophytes, antimicrobial, antibiofilm, *Staphylococcus aureus*, MRSA, natural products, bioactive compounds

## Abstract

The threat to public health resulting from the emergence of antimicrobial resistance (AMR) is ever rising. One of the major bacterial pathogens at the forefront of this problem is methicillin-resistant *Staphylococcus aureus*, or MRSA, for which there is a great need to find alternative treatments. One of the most promising alternatives is endophytic fungi, which were shown to produce a vast array of bioactive compounds, including many novel antibacterial compounds. In this study, two endophytic *Alternaria* sp., EL 24 and EL 35, were identified from the leaves of *Eremophila longifolia*. Ethyl acetate (EtOAc) extracts of their culture filtrates were found to inhibit both methicillin-sensitive *S. aureus* ATCC 25923 and MRSA strains M173525 and M180920. The activity of each extract was shown to be greatly affected by the growth medium, with considerable reductions in minimum inhibitory concentrations (MICs) and minimum bactericidal concentrations (MBCs) observed when tested in tryptic soy broth with glucose (TSBG) compared with Mueller–Hinton broth (MHB). Both extracts displayed significant (*p* ≤ 0.05) antibiofilm activity against all three *S. aureus* strains, the greatest of which was that of EL 35, which reduced biofilm formation by M180920 by 72%, while that of EL 24 resulted in a 57% reduction against ATCC 25923. Both extracts also disrupted established biofilms, of which the most effective was EL 35, which reduced the M180920 biofilm by 64%, while EL 24 also performed best against M180920, reducing biofilm by 54%. Gas chromatography–mass spectrometry (GC-MS) analysis of the EL 24 EtOAc extract revealed five known compounds. This study highlights the promise of endophytic fungi from Australian plants as a potential source of substances effective against important bacterial pathogens. Further understanding of the responsible compounds and their mechanisms could lead to the development of treatments effective against MRSA, as well as novel biofilm-resistant biomedical materials, contributing towards reducing the burden of AMR.

## 1. Introduction

Antimicrobial resistance (AMR) represents one of the greatest challenges facing global public health in the 21st century. In the most comprehensive study of the topic published to date, it was suggested that bacterial AMR was associated with approximately 4.95 million deaths globally in 2019, while 1.27 million people died as a direct result of drug-resistant infections [1]. It was estimated that without sufficient intervention, 10 million people could die around the globe annually by 2050 as a direct result of AMR, at an economic cost of around USD 100 trillion [2]. The problem of AMR has the potential to reverse the progress made by modern medicine in the treatment of infectious diseases, while also increasing the risks of other important medical interventions such as invasive surgeries, dialysis, and chemotherapy [3].

The Gram-positive bacterium *Staphylococcus aureus* is one of the major pathogens associated with AMR. In 2019, methicillin-resistant *S. aureus* (MRSA) infections resulted in the deaths of approximately 100,000 people worldwide [1], with high isolation rates reported in many countries around the globe [4,5]. Infections caused by MRSA are among the most common hospital-acquired infections, and are often associated with higher morbidity and mortality, as well as increased hospital stays and treatment costs [6]. MRSA are resistant to beta-lactam antibiotics such as penicillin, methicillin, and oxacillin, so treatment of MRSA infections is typically carried out using glycopeptides such as vancomycin and teicoplanin [7,8]. However, resistance to these drugs was also reported [9], while other antibiotics that are still effective, such as mupirocin and clindamycin, are only prescribed in the absence of other alternatives to reduce the risk of resistance development [8]. As such, there is a constant demand to find alternative solutions for the treatment and prevention of MRSA infections.

One of the most promising alternatives gaining a lot of attention in recent years is endophytes. Endophytes are symbiotic microorganisms that inhabit the inner tissues of plants without causing any signs of harm or disease and are widely recognised as an important source for the discovery of novel bioactive compounds [10]. Endophytic fungi, in particular, were reported to produce secondary metabolites with wide-ranging bioactivities, including antimicrobial, antifungal, antibiofilm, antiviral, anticancer, antioxidant, and antidiabetic activities [11]. While all plants are known to harbour endophytic fungi, plants with a history of medicinal use receive special attention, given that their medicinal properties may also be attributable to compounds produced by their fungal endophytes, with some compounds isolated from plants even found to be produced by endophytes present within their plant hosts [12,13,14]. For example, the discovery of a taxol-producing endophytic fungus *Taxomyces andreanae*, from the bark of *Taxus brevifolia*, the original plant source of this highly successful anticancer compound, sparked a global interest in endophyte natural products [15]. Similarly, the anticancer drug camptothecin, originally isolated from the Chinese medicinal plant *Camptotheca acuminata*, was subsequently isolated from *Fusarium solani*, an endophytic fungus found in the same plant [16]. Endophytic fungi previously demonstrated a capability to produce novel compounds displaying significant antimicrobial activity towards *S. aureus*, including MRSA, as well as a vast range of other bacterial species [17,18].

In this study, the aim was to investigate two endophytic fungi, EL 24 and EL 35, previously isolated by our group from the leaves of *Eremophila longifolia*, for their antibacterial and antibiofilm activities against *S. aureus* and MRSA. First, each isolate was identified through DNA sequencing. Next, ethyl acetate (EtOAc) extracts obtained from the fungal culture broths were screened for their inhibitory and bactericidal effects, while their ability to inhibit biofilm formation, as well as their ability to disrupt established biofilms, was also evaluated. Finally, chemical characterisation of the EL 24 extract was carried out by gas chromatography–mass spectrometry (GC-MS) analysis to determine the presence of antimicrobial components.

## 2. Results

### 2.1. Molecular Identification of Endophytic Fungi

Two endophytic fungi isolated from *E. longifolia* were selected for this study, based on previous antibacterial screening of 27 isolates from this plant, as well as 16 isolates from *Eremophila maculata*, during which the isolates EL 24 and EL 35 (Figure S1) displayed the most promising activity (Supplementary Materials Tables S1 and S2). Therefore, these isolates were chosen for further analysis, including their identification and a more detailed investigation of their antibacterial and antibiofilm activities. Molecular identification of EL 24 and EL 35 was carried out by sequencing the internal transcribed spacer (ITS) region of the ribosomal RNA (rRNA), the sequences of which were submitted to GenBank and given the accession numbers OR295218.1 (EL 24) and OR295219.1 (EL 35). Comparison of the obtained sequences with those in the National Centre for Biotechnology Information (NCBI) database using the Basic Local Alignment Search Tool (BLAST) identified both EL 24 and EL 35 as *Alternaria* sp.

### 2.2. Antibacterial Activity of Fungal Extracts

**Figure 1:**
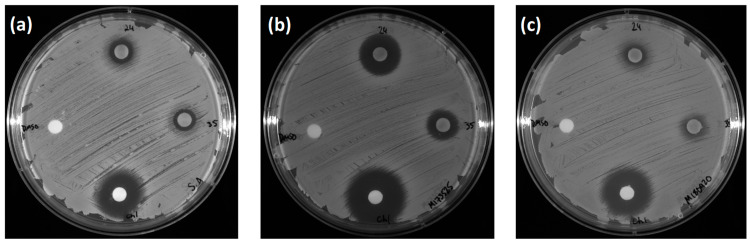
The disk diffusion assay revealed inhibition of (**a**) *S. aureus* ATCC 25923, (**b**) MRSA M173525 and (**c**) MRSA M180920. In each image, the effects of the EtOAc extract of EL 24 (top disk) and EL 35 (right disk) at 10 mg/mL are shown, as well as the positive control of chloramphenicol (bottom disk) at 3 mg/mL and the negative control of DMSO (left disk).

EtOAc extracts of EL 24 and EL 35 were assessed for antibacterial activity against three strains of *S. aureus* using a disk diffusion assay. As shown in Figure 1, the MRSA strain M173525 appeared to be the most susceptible strain to both EL 24 and EL 35, with inhibition zones of 16 and 12 mm, respectively (Figure 1b). For both extracts, a 9 mm inhibition zone was recorded against *S. aureus* ATCC 25923 (Figure 1a), while in the case of the MRSA strain M180920, EL 24 produced an inhibition zone of 8 mm, and EL 35 produced a 10 mm zone, although partial growth was still visible within this zone (Figure 1c).

The minimum inhibitory concentration (MIC) and the minimum bactericidal concentration (MBC) of the EtOAc extracts obtained from each of the two isolates were also evaluated against the three *S. aureus* strains, the results of which are summarised in Table 1 and Table 2, respectively. This assay was performed in two different growth media: Mueller–Hinton broth (MHB) and tryptic soy broth supplemented with 1% glucose (*w*/*v*) (TSBG). In the present study, both extracts displayed inhibitory activity as well as bactericidal activity towards all three strains of *S. aureus*. Overall, the EL 24 extract performed better than EL 35, having a lower MIC against each strain in both MHB and TSBG. The MBC values were generally two to four times greater than their respective MICs, again with those of EL 24 being lower than those of EL 35, except for two conditions. In the case of *S. aureus* ATCC 25923 grown in MHB, both EL 24 and EL 35 showed identical MBCs, while for MRSA M180920, the MBC of EL 35 was slightly lower than that of EL 24. In both cases, the MIC and MBC of EL 35 did not differ.

Interestingly, considerable differences were observed in the MIC and MBC values when tested in the different media, with all values being much lower when measured in TSBG compared to MHB. For EL 24, MIC values ranging from 0.4 to 0.8 µg/mL were recorded in TSBG, while those in MHB were 64- to 128-fold greater, ranging from 50 to 78 µg/mL. In TSBG, MBC values between 1.5 and 6.2 µg/mL were 25- to 128-fold lower than those in MHB, which ranged from 100 to 200 µg/mL. Similarly, for EL 35, MIC values were between 32- and 130-fold lower in TSBG than those in MHB, ranging from 1.2 to 4.9 µg/mL and 156 to 312 µg/mL, respectively. MBC values differed by 16- and 260-fold, ranging from 1.2 to 9.8 µg/mL in TSBG and from 156 to 625 µg/mL in MHB. In the case of the positive control, the antibiotic chloramphenicol, a reduction in the MIC values in TSBG compared with MHB was also seen, with MICs ranging from 0.4 to 1.6 µg/mL in TSBG, 4- to 16-fold lower than the MIC of 6.2 µg/mL recorded against all three strains in MHB. The bactericidal activity of chloramphenicol also increased considerably when TSBG was used. In MHB, all strains were able to grow from the highest concentration of chloramphenicol tested (100 µg/mL), whereas in TSBG, MBC values ranged from as low as 0.8 µg/mL against M180920 to 12.5 µg/mL against ATCC 25923. 

In general, all three *S. aureus* strains displayed similar susceptibility to the EtOAc extracts of EL 24 and EL 35. In the case of EL 24, the MIC in MHB was slightly lower against the two MRSA strains, at 50 µg/mL against each, compared with 78 µg/mL against ATCC 25923. The MBCs ranged from 100 to 200 µg/mL, the lowest of which was recorded against M180920. In TSBG, the MIC of EL 24 was lowest against M180920 at 0.4 µg/mL and was equal to or lower than that of chloramphenicol against each strain. A similar pattern was seen for the MBC of EL 24 in TSBG, in which the lowest value of 1.6 µg/mL was recorded against both MRSA strains, while all but M180920 performed at least on par with chloramphenicol. For EL 35, the MIC in MHB was lowest against ATCC 25923 and M180920, at 156 µg/mL, while the MBC of the same value was the lowest, recorded against ATCC 25923. In TSBG, the lowest recorded MIC and MBC was against M180920, both of which were 1.2 µg/mL.

### 2.3. Antibiofilm Activity of Fungal Extracts

**Figure 2:**
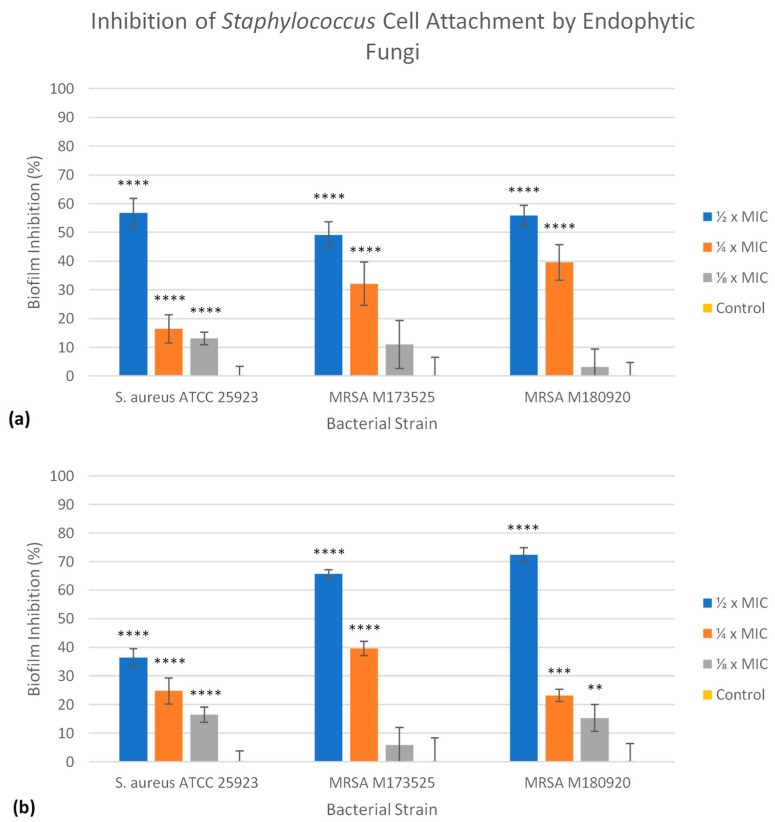
The EtOAc extracts of (**a**) EL 24 and (**b**) EL 35 were able to inhibit bacterial cell attachment on polystyrene when tested against three different strains of *S. aureus* at different concentrations. All values are calculated relative to the untreated control and displayed as the mean of all measurements, with error bars representing the standard deviation (SD). Statistical significance was calculated as test vs. untreated control, where ** = *p* ≤ 0.01, *** = *p* ≤ 0.001, and **** = *p* ≤ 0.0001.

The antibiofilm activity of the EtOAc extracts of both EL 24 and EL 35 was assessed against the three strains of *S. aureus*. For each extract, their ability to prevent the initial bacterial cell attachment on polystyrene was investigated, as was their ability to disrupt an established biofilm. In the case of the initial cell attachment assay, three different concentrations were tested: ½, ¼, and ⅛ × MIC. Since TSBG was used for the biofilm assays, the extract concentrations were based on the MIC results obtained using this medium. In this study, both EL 24 and EL 35 extracts were able to significantly (*p* ≤ 0.05) inhibit bacterial cell attachment and reduce the amount of biofilm formation to some degree at most concentrations tested. For EL 24 (Figure 2a), similar reductions in biofilm biomass were seen for all three *S. aureus* strains, ranging from 49 to 57%, when tested at ½ × MIC. At ¼ × MIC, EL 24 was more effective against M180920, with a 40% reduction in biomass, while reductions of 32 and 16% were recorded against M173525 and ATCC 25923, respectively. Smaller reductions ranging from 3 to 13% were also seen at ⅛ × MIC. As shown in Figure 2b, the EL 35 extract was found to be more effective than EL 24 against the two strains of MRSA when tested at ½ × MIC, reducing the amount of biofilm biomass by 72 and 66% of M180920 and M173525, respectively. However, the EL 35 extract was less effective against ATCC 25923, showing a 36% reduction. At ¼ × MIC, EL 35 was still able to reduce some biofilm formation, with reductions ranging from 23 to 40%, the highest being against M173525, while at ⅛ × MIC, smaller reductions ranging from 6 to 17% were recorded.

**Figure 3:**
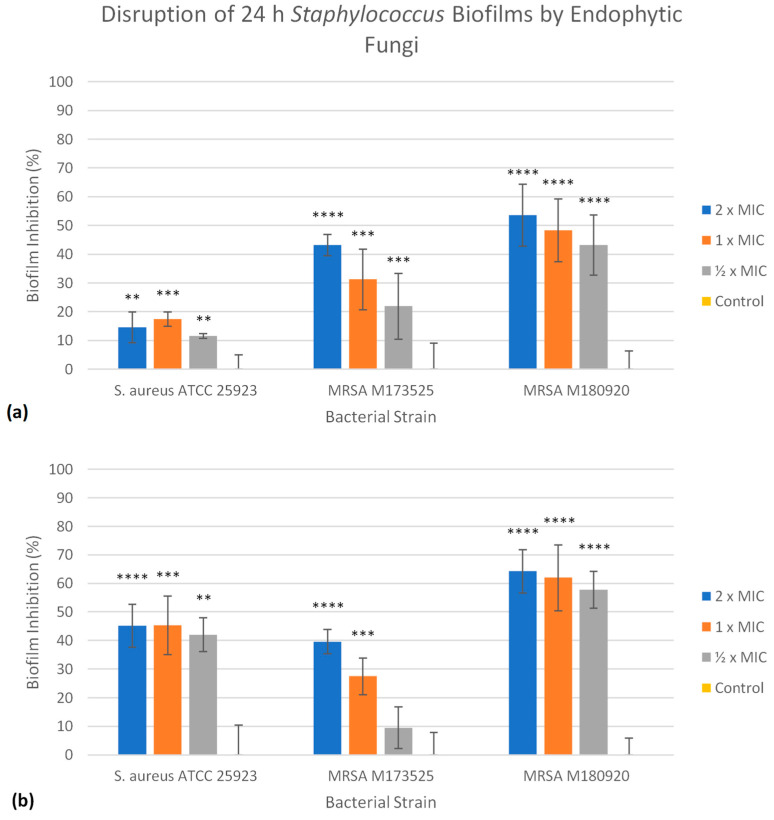
The EtOAc extracts of (**a**) EL 24 and (**b**) EL 35 were able to significantly disrupt the established biofilms formed on polystyrene by three different strains of *S. aureus* to different degrees. The greatest effect of each extract was recorded against M180920, with EL 24 and EL 35 reducing biofilm by up to 54 and 64%, respectively. All values are calculated relative to the untreated control and displayed as the mean of all measurements, with error bars representing the SD. Statistical significance was calculated as test vs. untreated control, where ** = *p* ≤ 0.01, *** = *p* ≤ 0.001, and **** = *p* ≤ 0.0001.

The ability of each extract to disrupt the established biofilms of all three *S. aureus* strains on polystyrene was analysed, again at three different concentrations, including 2, 1, and ½ × MICs. In this case, treatment with either EL 24 or EL 35 was able to significantly (*p* ≤ 0.05) reduce the biofilm biomass to some degree. As shown in Figure 3a, EL 24 was more effective against M180920 compared with the other strains, with biofilm reductions ranging from 43 to 54% at ½ and 2 × MIC, respectively. In contrast, the highest reduction in biomass seen for EL 24 against M173525 was 43% at 2 × MIC, falling to 22% at ½ × MIC, while lower reductions in biomass were recorded against ATCC 25923, ranging from 12 to 17%. As can be seen in Figure 3b, the EL 35 extract outperformed that of EL 24, except when tested against M173525. Similar to EL 24, EL 35 was most effective against M180920, with biofilm reductions ranging from 58 to 64% at ½ and 2 × MIC, respectively. The EL 35 extract was also far more effective against the ATCC 25923 strain, with the lowest reduction being 42% at ½ × MIC, up to 45% at both 1 and 2 × MIC. The effect of EL 35 on M173525 was similar to that of EL 24, albeit slightly lower, with biomass reductions ranging from 10% at ½ × MIC to 40% at 2 × MIC.

### 2.4. Gas Chromatography–Mass Spectrometry Analysis

**Figure 4:**
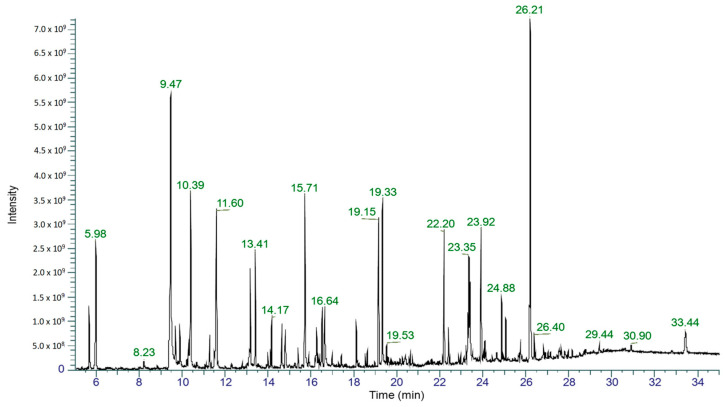
GC-MS analysis was carried out for the EtOAc extract of EL 24, the TIC of which shows numerous peaks.

**Figure 5:**
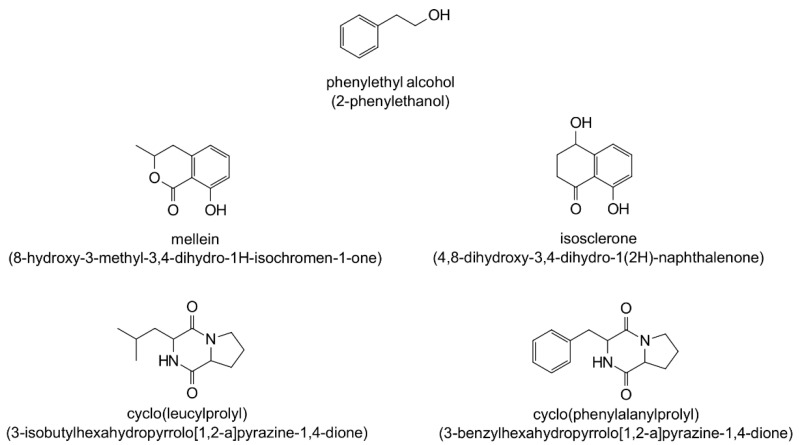
Chemical structure of compounds identified from the EtOAc extract of EL 24, showing both their common names along with their IUPAC names.

The EtOAc extract of EL 24 was selected for analysis by gas chromatography–mass spectrometry (GC-MS) to identify chemical constituents. The total ion chromatogram (TIC; Figure 4) of EL 24 shows numerous peaks, of which five were tentatively identified based on their match to compounds listed in the National Institute of Standards and Technology (NIST) Mass Spectral Library and Wiley Registry. The largest peak, with a retention time of 26.21 min, could not be identified in this case. Of the compounds that were identified in the extract, phenylethyl alcohol (RT = 9.47 min) was the most abundant, representing 12% of the total peak area, while other compounds, including mellein (RT = 15.71 min), isosclerone (RT = 16.64 min), cyclo(leucylprolyl) (RT = 19.15 min), and cyclo(phenylalanylprolyl) (RT = 23.40 min), were also detected in smaller amounts, each with peak areas less than 5%. These results are summarised in Table 3, while their chemical structures are shown in Figure 5.

## 3. Discussion

*E. longifolia* has a history of medicinal use by indigenous Australians, who used the plant for treating and preventing a range of ailments [19]. While endophytic fungi from *E. longifolia* were assessed for enzyme production and anticancer activity [20,21], the antimicrobial properties of these microorganisms were not investigated. In this study, the antibacterial activity of two endophytic isolates from *E. longifolia* was evaluated. These two isolates, EL 24 and EL 35, were identified as *Alternaria* sp. through DNA sequence analysis. Endophytic fungi belonging to this genus were previously isolated from *E. longifolia* [20], as well as being frequently isolated as an endophyte from many other plants [22,23,24].

In terms of *Alternaria*, extracts and isolated compounds obtained from endophytic fungi belonging to this genus were previously reported to possess a broad spectrum of antimicrobial activities [25,26]. In this study, EtOAc extracts of both EL 24 and EL 35 displayed promising inhibitory activity towards three different strains of *S. aureus*, including, importantly, the two methicillin-resistant strains. These findings are consistent with previous reports of antibacterial activity of EtOAc extracts from endophytic *Alternaria* isolates tested against *S. aureus*, such as those by Hamed et al. (2020) [27], who reported MIC values of 45 and 48 µg/mL for the extracts of two *Alternaria alternata* strains against *S. aureus* ATCC 6538-P. Mousa et al. (2021) [28] reported that the crude EtOAc extracts from an endophytic *A. alternata* and *Alternaria tenuissima* produced inhibition zones between 25 and 30 mm against *S. aureus* ATCC 6538P when tested by well diffusion. In another study, an *A. alternata* EtOAc extract produced MICs ranging from 100 to 900 µg/mL against 10 different clinical strains of *S. aureus* [29]. Previous reports indicated that *Alternaria* sp. can also produce compounds that could inhibit MRSA [30], while crude extracts also show activity against MRSA, although sometimes at much higher concentrations than those displayed by fungal extracts during the present study. For example, Techaoei et al. (2021) [31] reported that crude extracts of *A. alternata* inhibited the growth of MRSA, with MIC and MBC values of 4800 and 9600 µg/mL, respectively. On the other hand, Khiralla et al. (2016) [32] reported two *Alternaria* endophytes from which the EtOAc extracts displayed MICs of 125 and 250 µg/mL against MRSA, which is comparable to those displayed by EL 35, although still 2.5- to 5-fold higher than those displayed by EL 24. These findings suggest that endophytic *Alternaria* sp. isolated from *E. longifolia* represent a promising source of anti-MRSA compounds.

In this study, the antibacterial effects of each extract were analysed in two different growth media. The first, MHB, was selected due to its use as a standard medium for antimicrobial testing [33], while the second, TSBG, was selected based on its superior ability to support biofilm formation and growth by *Staphylococcus* sp. [34,35]. Considering that the biofilm assay was to be performed with concentrations based on the MIC of extracts, the MIC was also determined in TSBG. Interestingly, the antibacterial activity of both fungal extracts was dramatically affected by the medium in which they were tested, becoming much more potent in TSBG compared to MHB. A study by Hulankova (2022) [36] reported that the median MIC values of oregano essential oil against a range of bacteria was generally lower in TSB (474 µg/mL) than in MHB (616 µg/mL), with a similar but smaller effect seen for cinnamon essential oil. The author suggested that the higher proteinous content of MHB compared to TSB, as well as the starch content of MHB, possibly played a role in the observed effects through interactions between proteins and essential oil components. Indeed, several studies reported a reduction in MIC values of antimicrobial substances due to protein interactions [37,38,39], while starch was also shown to have a negative effect on the antimicrobial activity of essential oils derived from oregano and thyme [40]. The MIC of allicin, a compound extracted from garlic, was also shown to be media-dependent, resulting from its stability and availability in different media [41]. Other studies showed that the pH of the growth medium can have a significant effect on antimicrobial activity [42]; however, the pH of MHB and TSB used in this study is typically around pH 7.3 for both [43], suggesting that pH was not a factor in this case. Furthermore, Buyck et al. (2012) [44] showed that the MIC of the antibiotic azithromycin against *Pseudomonas aeruginosa* was much lower in eukaryotic cell culture medium compared to MHB, resulting from decreased expression of the *opr*M gene as well as increased permeability of the outer membrane. There are, however, no reports in the literature describing media-dependent MIC or MBC differences of the magnitudes observed in this study. While it seems likely that these effects are due to various interactions between extract and media components, or to differences in bacterial growth and gene expression in the different media, determination of the exact cause requires further investigation. Such findings highlight the importance of media choice when assessing antimicrobial activity, particularly when different assays are being performed.

Bacterial biofilms are surface-associated aggregates that can be composed of one or multiple species of bacteria that are encased in an extracellular polymeric matrix consisting of various biomolecules, such as polysaccharides, proteins, and extracellular DNA [45,46]. For the bacterial cells within the biofilm, transcriptional factors provide increased protection from external stressors, while the polymeric matrix also increases their ability to reduce such effects by providing a physical barrier [45,46]. In this state, bacterial cells display a much greater resistance to antimicrobial agents, at concentrations up to 1000-fold greater than those effective against planktonic cells [47]. In clinical settings, biofilms are of particular concern, as they are estimated to be responsible for around two thirds of nosocomial infections, contributing to both chronic infections and those associated with medical implants [46,48]. As such, there is a great need to discover novel compounds effective against bacterial biofilms, including compounds capable of inhibiting their formation as well as compounds that can eradicate established biofilms. Due to the recognition of *S. aureus* as a major biofilm-forming pathogen [49], in this study, EtOAc extracts of *Alternaria* isolates, EL 24 and EL 35, were assessed for their antibiofilm activity against three different strains of *S. aureus*. Both extracts were able to significantly (*p* ≤ 0.05) inhibit the bacterial cell attachment of all three *S. aureus* strains in a concentration-dependent manner, even at relatively low extract concentrations. For example, in the case of EL 24, a biofilm reduction between 49 and 57% was observed against the three bacterial strains at ½ × MIC, equating to concentrations of 0.4 µg/mL against *S. aureus* ATCC 25923 and MRSA M173525, and 0.2 µg/mL against MRSA M180920. In contrast, the EtOAc extract from an endophytic *Aspergillus* fungus was able to reduce biofilm formation of *S. aureus* NRRL B-767 by 80%, but at a concentration of 500 µg/mL [50]. Both EL 24 and EL 35 were also able to disrupt established biofilms of each strain to varying degrees, the best of which was EL 35 against MRSA M180920, with a 64% reduction in the biofilm biomass recorded, while the same strain was also the most susceptible to EL 24. Previous reports of endophytic fungal extracts disrupting *S. aureus* biofilms were published, such as that by Fathallah et al. (2019) [51], in which an EtOAc extract of an endophytic fungus, *Aspergillus amstelodami*, completely eradicated a *S. aureus* ATCC 25923 biofilm at concentrations > 62.5 µg/mL. A study by Jalil et al. (2021) [52] found that an EtOAc extract of an endophytic fungus, *Lasiodiplodia pseudotheobromae*, possessed antibiofilm activity against MRSA ATCC 33591 at concentrations > 100 µg/mL, yet stimulated biofilm production at concentrations between 10 and 60 µg/mL. Compared to such reports, the antibiofilm activity of crude EtOAc extracts of both EL 24 and EL 35 towards *S. aureus* and MRSA strains at concentrations < 1 µg/mL is quite noteworthy, warranting further investigation of the mechanism of action and the compounds responsible. The fact that EL 35 was found to be overall more effective than EL 24 at inhibiting and disrupting biofilms, while both the MICs and MBCs of EL 24 were consistently lower than those of EL 35, is also an interesting point. This suggests that different compounds are likely responsible for the different activities, or that various active components are produced at different concentrations by each isolate. This is, however, only speculation, and is the subject of on-going investigations.

Chemical characterisation of the EL 24 EtOAc extract by GC-MS resulted in the putative identification of five compounds, all of which are known natural products. Phenylethyl alcohol was previously identified as an *Alternaria* metabolite [53], and has known antimicrobial properties [54,55]. Due to its bacteriostatic properties, it is widely used as a preservative in pharmaceuticals and cosmetics [56]. Mellein is a phenolic compound and known fungal metabolite with broad-spectrum antimicrobial activity [57]. Although it was not previously reported as an *Alternaria* metabolite, the metabolic gene clusters responsible for its biosynthesis were identified in *A. alternata* [58]. Isosclerone, an isomer of mellein, is a tetralin compound and known *Alternaria* metabolite [59]; however, it was found to be inactive against *S. aureus* in a previous study [60]. Both cyclo(leucylprolyl) and cyclo(phenylalanylprolyl) are cyclic dipeptides that were previously reported as metabolites of *Alternaria* sp. [61]. Cyclo(leucylprolyl) was shown to be active against *Streptococcus mutans* and *Listeria monocytogenes*, while also demonstrating antibiofilm effects against the same bacteria [62,63]. Cyclo(phenylalanylprolyl) also showed broad spectrum antibacterial activity [64] and displayed synergistic activity with a range of antibiotics [65], as well as antibiofilm activity against *S. aureus*, although this occurred only at a concentration of 3 mg/mL [66], much greater than the concentration used in the current study. While this suggests that the activity displayed by the EtOAc extract of EL 24 is the result of several compounds, whether or not the identified compounds play any role is unknown at this stage. It is also likely that many other compounds are produced by this fungus, particularly those that lack the volatility required to be detected by GC-MS. The identification of other compounds produced by these endophytic fungi, and research into their antibacterial and antibiofilm activities are the subject of on-going investigations.

## 4. Materials and Methods

### 4.1. Molecular Identification of Endophytic Fungi

Genomic DNA of two endophytic fungi previously isolated from the Australian native plant, *E. longifolia*, was extracted from their mycelia, which was previously collected from liquid cultures grown in 250 mL of potato dextrose broth (PDB) for 2 weeks. The extraction was performed using a Quick-DNA™ Fungal/Bacterial Miniprep Kit (Zymo Research, Irvine, CA, USA) according to the instructions supplied by the manufacturer. For each fungal isolate, approximately 150 ng of the extracted DNA template was added to a PCR tube, along with 25 µL of MangoMix™ (Bioline, Eveleigh, Australia). Next, 1 µL of each primer, ITS1 (5′-TCC GTA GGT GAA CCT GCG G-3′) and ITS4 (5′-TCC TCC GCT TAT TGA TAT GC-3′), was added to the reaction mix. The final volume of each was then made up to 50 µL using MilliQ purified water. To amplify the target region, this reaction mixture was then subjected to PCR using a thermocycler consisting of 1 denaturation cycle at 95 °C for 5 min, followed by 35 cycles of 95 °C for 30 s, 58 °C for 45 s, and 72 °C for 60 s for the annealing and extension stage and then 1 cycle at 72 °C for 7 min for the final extension. Following PCR, the products were analysed by gel electrophoresis in 1% (*w*/*v*) agarose gel to confirm that the intended target region was successfully amplified and that no unwanted bands were present. The PCR products were then purified using an ISOLATE II PCR and Gel Kit (Bioline, Eveleigh, Australia) according to the instructions provided by the manufacturer. All DNA samples to be sequenced were then prepared as per the guidelines provided by the Australian Genome Research Facility (AGRF; Melbourne, Australia), to which the samples were then sent for sequencing. All sequencing data were then analysed using BLAST available on the NCBI GenBank database, then submitted to NCBI.

### 4.2. Preparation of Fungal Extracts

As part of this study, two fungal endophytes previously isolated from the leaves of *E. longifolia*, of which plant material was supplied by the Canopus Corporation (Byrock, Australia), were investigated for their antibacterial and antibiofilm activity. To prepare extracts for screening, each fungal isolate was first sub-cultured on PDA plates and incubated at 28 °C for 5 to 7 days. This actively growing culture was then used to inoculate 250 mL of PDB in a 500 mL conical flask, which was subsequently incubated for 14 days at 28 °C with shaking at 150 rpm. Following incubation, the fungal biomass was separated from the fermentation broth by vacuum filtration through Whatman filter paper No. 1. The biomass was discarded, while the filtrate was extracted twice with equal volumes of EtOAc. The solvent was then removed under a vacuum at 40 °C using a rotary evaporator (BUCHI, Essen, Germany) until only a small amount remained, at which point the remaining extract was transferred to 1.5 mL microcentrifuge tubes that were then placed in a vacuum concentrator (Christ, Osterode am Harz, Germany) at 40 °C to remove all remaining solvent. The dried extracts were weighed and then stored at −20 °C until required.

### 4.3. Bacterial Cultures

Extracts were tested for their antibacterial and antibiofilm activity against *S. aureus* (ATCC 25923), obtained from the Department of Chemistry and Biotechnology at Swinburne University of Australia. They were also tested against two clinical isolates of methicillin-resistant *S. aureus*: strains M173525 and M180920. For experiments, bacteria were grown on Mueller–Hinton agar (MHA) plates for 24 h at 37 °C. From these plates, liquid cultures were prepared by inoculating either MHB or TSBG, which was then also incubated for 24 h at 37 °C.

### 4.4. Disk Diffusion Assay

Antibacterial activity of the fungal EtOAc extracts was evaluated using the agar disk diffusion method described by Balouiri, Sadiki, and Ibnsouda (2016) [67], with minor modifications. First, overnight cultures prepared in MHB were diluted with sterile MHB until the optical density was equal to that of the 0.5 McFarland standard, as measured spectrophotometrically (OD_600_ = 0.08–0.1), giving an approximate cell concentration of 1 × 10^8^ colony-forming units (CFU)/mL. Next, the surfaces of MHA plates were inoculated with each bacterial solution using a sterile cotton swab. Paper disks 6 mm in diameter were loaded with 10 µL of each test sample, along with positive and negative controls. For this purpose, fungal extracts were dissolved to 10 mg/mL in DMSO. Chloramphenicol (3 mg/mL; Sigma-Aldrich, Macquarie Park, Australia) was used as a positive control, while DMSO was used as a negative control. The loaded disks were then placed on the inoculated agar surface, after which the plates were incubated overnight at 37 °C. Finally, inhibition zones were measured and reported to the nearest millimetre. All tests were performed in duplicate.

### 4.5. Determination of MIC and MBC

The MIC and the MBC of each EtOAc extract was evaluated using the broth microdilution method described by the European Committee on Antimicrobial Susceptibility Testing (EUCAST) [33]. Briefly, to prepare the bacterial inoculum, overnight cultures of each bacterium were prepared in MHB, as well as TSBG. Each culture was then adjusted to match the turbidity of the 0.5 McFarland standard, which was then further diluted 1:20 in their respective media to give a cell concentration of 5 × 10^6^ CFU/mL. Stock solutions of fungal EtOAc extracts were prepared in DMSO, of which 100 µL was added to the first well of a flat-bottomed polystyrene 96-well plate (SPL Life Sciences, Pocheon, Republic of Korea). In wells 2 to 12 of the plate, 50 µL of sterile MHB or TSBG was added. Next, a serial dilution of each test sample was prepared by taking 50 µL from the first well and transferring it to the second well of the corresponding row, mixing the solution by pipetting. This process was repeated, taking 50 µL from well 2 and transferring to well 3, then 3 to 4, and so on, while the final 50 µL from well 12 was discarded. Next, 40 µL of additional media was added, followed by 10 µL of the above bacterial solution, giving a final volume of 100 µL in each well with a bacterial cell concentration of 5 × 10^5^ CFU/mL. For controls, chloramphenicol (200 µg/mL) was included as a positive control, while DMSO was used as a negative control. Wells containing only media were also included to ensure sterility. The plates were then incubated for 20–24 h at 37 °C, after which they were inspected for growth. The MIC was determined as the lowest concentration to completely prevent bacterial growth as detected visually.

The MBC of each sample was also evaluated. This was accomplished by plating aliquots of 10 µL from each well containing no growth onto MHA plates and incubating overnight at 37 °C. The MBC was then determined as the lowest concentration at which no bacterial growth could be observed on the agar plate. All tests were performed in triplicate.

### 4.6. Antibiofilm Activity

The antibiofilm properties of each fungal EtOAc extract were then assessed against each bacterial strain. First, these samples were tested for their ability to prevent the initial cell attachment and subsequent biofilm formation using the crystal violet (CV) staining method described by Stepanović et al. (2007) [34] and Jadhav et al. (2013) [68], with some modifications. The samples were then also tested for their ability to disrupt established biofilms, again using CV staining, according to the methods described by Stepanović et al. (2007) [34] and Skogman, Vuorela, and Fallarero (2016) [69], with modifications.

#### 4.6.1. Inhibition of Cell Attachment

To prepare bacterial inoculums, each strain was first grown overnight on MHA plates at 37 °C. The following day, a single colony of each was used to inoculate approximately 3 to 4 mL of TSBG in a sterile polystyrene tube, then grown again overnight at 37 °C. After incubation, tubes were vortexed for 1 min and then adjusted to match the turbidity of the 0.5 McFarland standard using sterile TSBG. This solution was then further diluted 1:100 in sterile TSBG. For each fungal EtOAc extract, three different concentrations were tested, including ½, ¼, and ⅛ × MICs. In this assay, stock solutions of each extract were prepared at 50× their final desired concentration in DMSO. In a 96-well polystyrene plate (SPL Life Sciences, Republic of Korea), 4 µL of extract was added for each test sample, followed by 196 µL of the diluted bacterial suspension. A positive growth control (100% biofilm) was also included, containing only 200 µL of the bacterial suspension, as was a negative growth control (0% biofilm), containing 200 µL of TSBG. The plate was then incubated for 24 h at 37 °C. After incubation, culture medium was gently removed by pipetting, and the remaining biofilms were washed twice with sterile room temperature phosphate-buffered saline (PBS). The plate was then blotted dry on a paper towel before being placed in an oven at 60 °C for 1 h to fix the biofilms. Next, 190 µL of 0.02% CV was added to each well and incubated at room temperature for approximately 10 min, after which the CV was gently removed by pipetting. All wells were then rinsed three times with room temperature PBS, and finally under gently running distilled water until no more stain was visible in washings. The bound stain was then dissolved by adding 150 µL of 33% acetic acid to each well, then leaving the plate at room temperature until all the stain dissolved. The absorbance of each well was then measured at 595 nm using a microplate reader (POLARstar Omega, BMG Labtech, Ortenberg, Germany). All measurements were performed in at least triplicate, with all data reported as an average of these results. The inhibition of biofilm formation was then calculated relative to the controls according to the equation:

$$\mathrm{Percentage}\;\mathrm{inhibition}=100-\left(\frac{{\mathrm{Abs}}_{595}\;\mathrm{experimental}\;\mathrm{well}\;\mathrm{with}\;\mathrm{extract}}{{\mathrm{Abs}}_{595}\;\mathrm{control}\;\mathrm{well}\;\mathrm{with}\;\mathrm{no}\;\mathrm{extract}}\right)\times 100.$$

#### 4.6.2. Eradication of Established Biofilms

In order to test the effects of each fungal extract against established biofilms, the bacterial inoculum was prepared in the same way as the previous assay, whereby an overnight culture was adjusted to match the 0.5 McFarland standard and then diluted 1:100 in TSBG. For this assay, each fungal extract was tested at three concentrations, including 2, 1, and ½ × MICs, for which 50× stock solutions were again prepared in DMSO. In a 96-well polystyrene plate (SPL Life Sciences, Republic of Korea), 200 µL of the diluted bacterial suspension was added to each test well. As with the previous assay, positive and negative growth controls, containing 200 µL of bacterial suspension and 200 µL of TSBG, respectively, were also included. The plate was then incubated for 24 h at 37 °C. Following incubation, the culture medium was carefully removed by pipette and the wells washed twice with sterile room temperature PBS. For each test sample, 4 µL of fungal extract was added to wells, followed by 196 µL of fresh sterile TSBG, while for the control wells, 200 µL of TSBG was added. The plate was then incubated for a further 24 h at 37 °C. From this point on, the identical procedure to that performed in the cell attachment assay was carried out. Wells were washed, stained with CV, dissolved in 33% acetic acid, and the absorbance measured at 595 nm. All measurements were performed in at least triplicate and reported as the average, while the biofilm inhibition was calculated the same as previous.

### 4.7. GC-MS Analysis of the EL 24 EtOAc Extract

The EtOAc extract obtained from the culture filtrate of EL 24 grown in PDB was dissolved in dichloromethane and analysed by GC-MS. A Thermo Scientific TSQ 8000 TRACE 1310 GC mass spectrometer was used to capture mass spectra, using electron ionisation in the positive ion mode and a 70 eV ionisation energy. A SGE SOLGEL-1MS column (30 m × 0.25 mm ID, 0.25 µm film thickness) was used to carry out the gas chromatography with the following settings: a temperature program of 50 °C for 2 min, followed by heating at 25 °C/min until 300 °C, where the temperature was held for 3 min with a split injection. A split ratio of 10 was used, while the injector temperature and transfer line temperature were set at 300 °C, using high-purity helium with a 1 mL/min flow rate as the carrier gas. Peak picking and integration were performed by the automated Avalon algorithm in Freestyle 1.8 SP2 (Thermo Scientific, Scoresby, Australia), with the minimum threshold for peak integration set at 0.2% of total peak area. Retention indices were calculated manually via a linear calibration curve created from C8-C20 standard alkane mix run at the same time as the sample. Peaks were identified by comparison with the NIST 2017 Mass Spectral Library and Wiley Registry.

### 4.8. Statistical Analysis

Statistical analysis was carried out using Microsoft Excel (*t*-test) to determine the ability of EtOAc extracts of two endophytic fungi to inhibit the biofilm formation and disrupt established biofilms of *S. aureus* and MRSA. All data are presented as the mean of all replicates, with error bars representing the SD. A *p*-value equal to or less than 0.05 was considered statistically significant.

## 5. Conclusions

In conclusion, this study investigated the potential of two endophytic fungi isolated from *E. longifolia* and identified as *Alternaria* sp. to inhibit the growth of *S. aureus* and MRSA, including their ability to inhibit biofilm formation. As a result, it was confirmed that EtOAc extracts derived from both endophytes were inhibitory towards all three bacterial strains. Interestingly, the inhibitory effect was found to be highly media dependent. MICs ranging from 50 to 78 µg/mL and from 156 to 312 µg/mL were recorded in MHB for EL 24 and EL 35, respectively. However, when measured in TSBG, MICs dramatically reduced, ranging from 0.4 to 0.8 µg/mL for EL 24, and from 1.2 to 4.9 µg/mL for EL 35. A similar effect was seen for the MBCs, with those of EL 24 ranging from 100 to 200 µg/mL in MHB, falling from 1.6 to 6.2 µg/mL in TSBG, while for EL 35, MBCs between 156 and 625 µg/mL were recorded in MHB, dropping to between 1.2 and 9.8 µg/mL in TSBG. Each extract also showed significant antibiofilm activity against all three strains, reducing biofilm formation on polystyrene by preventing cell attachment. In this case, EL 35 displayed a greater effect, with the best result seen against MRSA strain M180920, with a 72% reduction in biofilm formation, while *S. aureus* ATCC 25923 was the most susceptible to EL 24, with a 57% reduction in the biofilm formed. Both extracts also displayed an ability to disrupt established biofilms, with EL 35 again performing better than EL 24. In both cases, M180920 was the most susceptible, with a 64 and 54% reduction in biofilm biomass recorded in the presence of EL 35 and EL 24, respectively. Five known metabolites were identified in the EL 24 extract by GC-MS analysis, some of which possess known antimicrobial activities, suggesting that multiple compounds played a role in the observed effects. Work is on-going to identify other compounds produced by these endophytes and to determine their antibacterial and antibiofilm properties.

## Figures and Tables

**Table 1:** The MIC of EtOAc extracts obtained from isolates EL 24 and EL 35 were determined in both MHB and TSBG, against *S. aureus* ATCC 25923 and two strains of MRSA, including M173525 and M180920. The antibiotic chloramphenicol was used as a positive control. The values shown are all in µg/mL.

Sample	ATCC 25923		M173525		M180920	
	MHB	TSBG	MHB	TSBG	MHB	TSBG
**EL 24**	78	0.8	50	0.8	50	0.4
**EL 35**	156	4.9	312	2.4	156	1.2
**Chloramphenicol**	6.2	1.6	6.2	0.8	6.2	0.4

**Table 2:** The MBC of the EL 24 and EL 35 extracts was also determined against the same strains in both media. All values are shown in µg/mL.

Sample	ATCC 25923		M173525		M180920	
	MHB	TSBG	MHB	TSBG	MHB	TSBG
**EL 24**	156	6.2	200	1.6	100	1.6
**EL 35**	156	9.8	625	2.4	312	1.2
**Chloramphenicol**	>100	12.5	>100	1.6	>100	0.8

**Table 3:** GC-MS was used to analyse the chemical composition of the crude EtOAc extract of EL 24. Compounds were identified by comparison with the NIST Mass Spectral Library and Wiley Registry, with those identified listed along with their retention times (RT), retention indices, relative peak area, match factor, molecular weight, and chemical formula.

RT (min)	Chemical Formula	Common Name (IUPAC Name)	Peak Area (%)	Molecular Weight	Retention Index	Match Factor (/1000)
9.47	C_8_H_10_O	Phenylethyl alcohol (2-phenylethanol)	12	122.16	1100	927
15.71	C_10_H_10_O_3_	Mellein (8-hydroxy-3-methyl-3,4-dihydro-1H-isochromen-1-one)	4.4	178.18	1570	844
16.64	C_10_H_10_O_3_	Isosclerone (4,8-dihydroxy-3,4-dihydro-1(2H)-naphthalenone)	1.8	178.18	1640	888
19.15	C_11_H_18_N_2_O_2_	Cyclo(leucylprolyl) (3-isobutylhexahydropyrrolo[1,2-a]pyrazine-1,4-dione)	3.8	210.27	1820	809
23.40	C_14_H_16_N_2_O_2_	Cyclo(phenylalanylprolyl) (3-benzylhexahydropyrrolo[1,2-a]pyrazine-1,4-dione)	2.4	244.29	2140	883

## Data Availability

Data are available upon reasonable request to the corresponding author.

## Supplementary Materials

The following supporting information can be downloaded at: https://www.mdpi.com/article/10.3390/antibiotics12091459/s1, Table S1: Antibacterial activity of endophytic fungi from *E. longifolia* and *E. maculata*; Table S2: Antibacterial activity of EtOAc extracts of endophytic fungi from *E. longifolia*; Figure S1: Cultures of EL 24 and EL 35.
