# Supplementary material for: Antibacterial and Antibiofilm Activity of Endophytic Alternaria sp. Isolated from Eremophila longifolia
Source: Antibiotics (Basel). 2023 Sep 19;12(9):1459. doi: 10.3390/antibiotics12091459 (PMC10525891; doi:10.3390/antibiotics12091459)
Supplement: Supplementary file 1 [file antibiotics-12-01459-s001.zip › Supplementary Materials.pdf]

Supplementary Materials

Table S1: Agar disk diffusion assay results using crude aqueous extracts obtained from fermentation broth filtrates of a range of endophytic fungi. Extracts were tested at 10 μL per disk against the pathogenic bacteria listed, and the zone of inhibition is given in mm. EL = *E. longifolia*, EM = *E. maculata*. N/A = not assessed, * = partial inhibition.

Sample	Zone of inhibition (mm) against				
	B. cereus	*E. coli*	*S. aureus*	*S. iniae*	*V. parahaemolyticus*
EL 1	-	-	-	-	-
EL 2	-	N/A	N/A	-	N/A
EL 3	N/A	-	-	9	-
EL 4	N/A	-	-	-	-
EL 5	-	-	-	-	-
EL 6	-	-	-	-	-
EL 7	-	-	-	-	-
EL 9	7	-	9	11	8
EL10	7	-	9	11	8
EL 11	-	-	-	-	-
EL 12	-	-	-	-	-
EL 13	-	-	-	-	-
EL 14	-	-	-	-	-
EL 15	-	-	-	-	-
EL 16	-	-	-	-	-
EL 17	-	-	-	-	-
EL 18	7	-	7	7	8
EL 19	-	-	11	11	-
EL 21	7	-	7	8	-
EL 22	-	-	-	-	-
EL 23	N/A	-	-	N/A	-
EL 24	16	N/A	13	13	-
EL 29	-	-	-	N/A	-
EL 32	-	-	-	-	-

EL 33	-	-	-	-	-
EL 35	12	-	13	11	7
EL 36	10	-	13	N/A	7
EM 1	-	-	-	-	-
EM 2	-	-	-	-	12*
EM 3	-	-	-	-	18*
EM 4	-	-	-	-	16*
EM 5	-	-	-	-	13*
EM 6	-	-	-	-	-
EM 7	-	-	-	-	14*
EM 8	-	-	-	-	-
EM 9	-	-	-	-	-
EM 10	-	-	-	-	-
EM 11	-	-	-	-	-
EM 12	-	-	-	-	-
EM 13	-	N/A	N/A	N/A	N/A
EM 14	9	N/A	N/A	N/A	N/A
EM 15	-	N/A	N/A	N/A	N/A
EM 16	9	N/A	N/A	N/A	N/A

Table S2: Agar disk diffusion results of ethyl acetate extracts obtained from selected endophytic isolates. All extracts were dissolved in DMSO and tested at 10 μL per disk, and the zone of inhibition measured to the nearest mm are shown for each. EL = *E. longifolia*, Chl = chloramphenicol (3 mg/mL), Amp = ampicillin (1 mg/mL), N/A = not assessed, * = partial inhibition.

**Sample**	**Zone of inhibition against (mm)**				
	B. cereus	*E. coli*	*S. aureus*	*S. iniae*	*V. parahaemolyticus*
EL 9	9	-	7	19	-
EL 19	14	-	16	20	-
EL 24	15	-	16	16	8
EL 35	21	-	17	19	7
EL 36	-	-	7*	8	-

Chlor	24	25	N/A	26	28
Amp	N/A	N/A	20	N/A	N/A

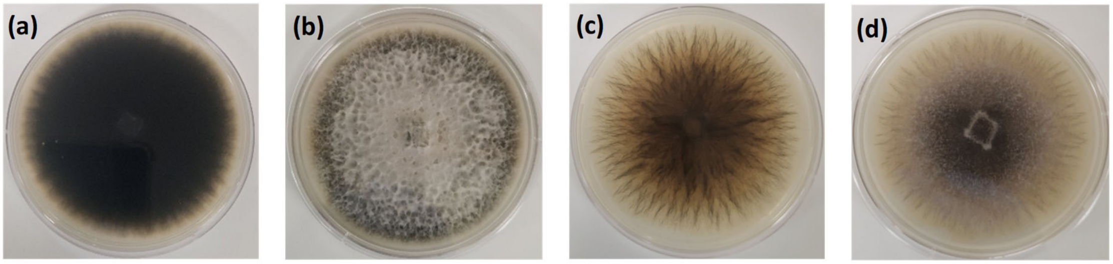

Figure S1: Endophytic fungal cultures grown for 7 days on potato dextrose agar (PDA). The images show EL 24 and EL 35 from the bottom (a and c, respectively) and top (b and d, respectively) of the plates.
